# Prenatal exposure to wildfire fine particulate matter and fetal growth outcomes in the United States: Findings from the ECHO Cohort

**DOI:** 10.1097/EE9.0000000000000526

**Published:** 2026-09-09

**Authors:** Allison R. Sherris, Logan C. Dearborn, Dana E. Goin, Christine T. Loftus, Adam A. Szpiro, Joan A. Casey, Sindana D. Ilango, Jyoti Angal, Deborah H. Bennett, Miatta A. Buxton, Carlos A. Camargo, Kecia N. Carroll, Marissa L. Childs, Camille Cioffi, Lisa A. Croen, Dana Dabelea, Stephanie M. Eick, Shohreh F. Farzan, Assiamira Ferrara, Erika Garcia, Alison Gemmill, Frank Gilliland, Rima Habre, Irva Hertz-Picciotto, Alison E. Hipwell, Deborah Hirtz, Margaret R. Karagas, Daphne Koinis-Mitchell, Amii M. Kress, Leslie D Leve, Donghai Liang, Kristen Lyall, Lacey A. McCormack, Cindy T. McEvoy, Hooman Mirzakhani, Rachel Morello-Frosch, Zhongzheng Niu, Thomas G. O’Connor, Alicia K. Peterson, Rebecca J. Schmidt, Catherine J. Karr, Amy M. Padula

**Affiliations:** a Department of Environmental and Occupational Health Sciences, University of Washington, Seattle, Washington; b Mailman School of Public Health, Columbia University, New York, New York; c Department of Biostatistics, University of Washington, Seattle, Washington; d Department of Environmental and Occupational Health Sciences and Department of Epidemiology, University of Washington, Seattle, Washington; e Department of Public Health Sciences, University of California, Davis, Davis, California; f Department of Pediatrics, Avera Research Institute, University of South Dakota School of Medicine, Sioux Falls, South Dakota; g Department of Epidemiology, University of Michigan, Ann Arbor, Michigan; h Department of Emergency Medicine, Massachusetts General Hospital, Harvard Medical School, Boston, Massachusetts; i Pediatrics and Environmental Medicine, The Icahn School of Medicine at Mount Sinai School, New York, New York; j Prevention Science Institute, University of Oregon, Eugene, Oregon; k Division of Research, Kaiser Permanente Northern California, Kaiser Permanente, Pleasanton, California; l Lifecourse Epidemiology of Adiposity and Diabetes (LEAD) Center, University of Colorado Anschutz Medical Campus, University of Colorado School of Medicine, Aurora, Colorado; m Gangarosa Department of Environmental Health, Rollins School of Public Health, Emory University, Atlanta, Georgia; n Department of Population and Public Health Sciences, University of Southern California, Los Angeles, California; o Division of Research, Kaiser Permanente Northern California, Kaiser Permanente, Pleasanton, California; p Department of Population, Family and Reproductive Health, Johns Hopkins University Bloomberg School of Public Health, Baltimore, Maryland; q Department of Population, Family and Reproductive Health, University of Southern California, Los Angeles, California; r Department of Psychiatry, Psychology, and Clinical & Translational Science, University of Pittsburgh, Pittsburgh, Pennsylvania; s Department of Neurological Sciences and Pediatrics, University of Vermont School of Medicine, Burlington, Vermont; t Department of Epidemiology, Geisel School of Medicine, Dartmouth College, Hanover, New Hampshire; u Department of Pediatrics, Brown University, Providence, Rhode Island; v Department of Epidemiology, Johns Hopkins University Bloomberg School of Public Health, Baltimore, Maryland; w Gangarosa Department of Environmental Health, Rollins School of Public Health, Emory University, Atlanta, Georgia; x AJ Drexel Autism Institute, Drexel University, Philadelphia, Pennsylvania; y Avera Research Institute, Avera McKennan Hospital & University Health Center, Sioux Falls, South Dakota; z Department of Pediatrics, Oregon Health & Science University, Portland, Oregon; aa Channing Division of Network Medicine, Brigham and Women’s Hospital, Harvard Medical School, Boston, Massachusetts; bb Department of Environmental Science, Policy and Management, School of Public Health, University of California, Berkeley, Berkeley, California; cc Department of Population and Public Health Science, University of Southern California, Los Angeles, California; dd Departments of Psychiatry, Neuroscience, Obstetrics and Gynecology, University of Rochester, Rochester, New York; ee Division of Research, Kaiser Permanente Northern California, Pleasanton, California; ff Department of Public Health Sciences, MIND Institute, University of California, Davis; Davis, California; gg Departments of Pediatrics and Environmental and Occupational Health Sciences, University of Washington, Seattle, Washington; hh Department of Obstetrics, Gynecology & Reproductive Sciences, University of California, San Francisco, San Francisco, California.

**Keywords:** Air pollution, Birth weight, Birth weight for gestational age, Fetal growth, LGA, Pregnancy, SGA, Smoke, Wildfire

## Abstract

**Background:**

Increasing exposure to wildfire fine particulate matter (PM_2.5_) may pose unique risks to fetal development. We assessed associations between prenatal wildfire PM_2.5_ exposure and fetal growth outcomes, including birth weight at term, birth weight for gestational age *z*-scores, small for gestational age, and large for gestational age in the U.S. Environmental Influences on Child Health Outcomes Cohort.

**Methods:**

The study included 20,034 births (2006–2020) from 30 cohort sites nationwide. We assigned daily wildfire PM_2.5_ at census-tract resolution and summarized average concentrations and the number of smoke days exceeding multiple intensity thresholds across pregnancy. We used covariate-adjusted mixed-effects models to estimate overall associations and distributed lag models and trimester-specific analyses to evaluate critical exposure windows across gestation, and we examined potential effect modification by US region, fetal sex, maternal race, and neighborhood poverty.

**Results:**

Pregnancy wildfire exposure metrics, including mean prenatal PM_2.5_ and the number of smoke days, were not associated with study outcomes. Distributed lag and trimester-specific models did not show consistent sensitive windows of exposure associated with birth weight at term, birth weight for gestational age *z*-scores, or small for gestational age. A positive association was observed between higher early-pregnancy wildfire exposure and large for gestational age (odds ratio = 1.015 per smoke day, 95% confidence interval: 1.001, 1.029), though effect estimates were small in magnitude. No consistent effect modification was identified.

**Conclusion:**

In this nationwide cohort, wildfire PM_2.5_ showed limited associations with fetal growth outcomes.

**What this study adds**

This study provides nationwide evidence on prenatal exposure to wildfire-specific particulate matter (PM_2.5_) and fetal growth outcomes using data from a large, prospective US birth cohort. Leveraging 20,034 births across 30 cohort sites in the ECHO Cohort, nationwide wildfire smoke exposure estimates, and detailed longitudinal data, this study addresses important gaps in understanding the reproductive health impacts of increasing wildfire activity. The geographic diversity, prospective design, and evaluation of sensitive exposure windows make this work well suited to the readership of *Environmental Epidemiology*.

## Introduction

Wildfires pose an increasing threat given the increasing occurrence and severity of global wildfire activity over recent decades.^1–3^ Widespread population exposure to fine particulate matter (PM_2.5_) is of particular concern, as wildfire PM_2.5_ has distinct physicochemical properties that may lead to higher toxicity relative to other sources of ambient PM_2.5._^4,5^ Compared with continued decline from other sources, concentrations of wildfire PM_2.5_ are also growing in magnitude across the United States.^6^ Exposure to PM_2.5_ from wildfire smoke has been implicated as a risk factor for a variety of adverse health outcomes, including respiratory, cardiovascular, and reproductive health.^1,7^ However, the impact of prenatal wildfire PM_2.5_ exposure on fetal growth and infant birth weight remains unclear despite biological plausibility.

Pregnant individuals and the developing fetus may be sensitive to impacts from wildfire PM_2.5_ due to maternal physiological changes during pregnancy, such as increased respiratory rate and cardiac output, and rapid fetal growth and development.^7^ As evidenced by studies of total PM_2.5_, exposure in pregnancy may impact maternal health outcomes including metabolic and hypertensive disorders,^8–10^ oxidative stress,^11,12^ systemic inflammation,^12,13^ placental inflammation and dysfunction,^14,15^ and epigenetic regulation,^16^ which are biological pathways implicated in the etiology of fetal growth abnormalities.^17–19^ However, these studies largely focused on ambient air pollution in general. Research suggests that wildfire PM_2.5_ toxicity is elevated relative to ambient PM_2.5_ from other sources due to differences in chemical composition, oxidative potential, and size distribution.^4,5,20^

There is suggestive evidence linking prenatal exposure to overall ambient PM_2.5_ with decreased birth weight and small for gestational age (SGA).^21,22^ These outcomes and related fetal growth restriction are associated with challenges including respiratory issues, feeding difficulties, and increased susceptibility to infection, as well as long-term risk of neurodevelopmental impairment and chronic cardiometabolic conditions such as hypertension.^23,24^ All-source ambient PM_2.5_ exposure during pregnancy has also been associated with outcomes related to fetal overgrowth, including macrosomia (>4,000 g) and large for gestational age (LGA).^25,26^ In the short term, these outcomes are associated with increased risks of birth trauma such as shoulder dystocia, metabolic abnormalities including hypoglycemia, respiratory distress, and other neonatal morbidities.^17,27^ Over the long term, infants born LGA or macrosomic have an elevated risk of childhood obesity, type 2 diabetes, and cardiovascular disease.^28^

Emerging literature provides evidence for an association between wildfire PM_2.5_ exposure and increased risk of low birth weight (<2,500 g) or decreased birth weight,^29–32^ with some evidence for a link to LGA and increased birth weight and/or birth weight for gestational age (BWGA).^33–35^ However, studies to date have focused on specific states^31,36^ or localities,^32,33^ with a few encompassing regions of the United States.^37,38^ Prior studies have also used varied exposure assessment methods and relied on administrative records that lack comprehensive data on important sociodemographic confounders and maternal residential history.^29^ Finally, research has largely focused on the health effects of average wildfire PM_2.5_ across pregnancy,^36,37,39^ despite the episodic nature of wildfire PM_2.5_ exposure relative to other ambient PM_2.5_.

There are also uncertainties regarding population vulnerability and susceptibility to wildfire smoke. The composition of wildfire smoke, behavioral responses to wildfire events, housing trends, environmental co-exposures, and outcome trends may vary geographically and by household income, leading to regional differences in associations.^40^ Prior studies have also identified racial disparities in the health effects of wildfire PM_2.5_, including for adverse birth outcomes.^41,42^ Characterizing at-risk subgroups, as well as the role of smoke exposure intensity, duration, and timing during pregnancy, can guide health promotion efforts for pregnant individuals during wildfire events.

Building on our earlier work in the ECHO Cohort identifying an association between wildfire PM_2.5_ and preterm birth,^43^ we assessed whether wildfire PM_2.5_ exposures during pregnancy were associated with the outcomes of birth weight at term, BWGA *z*-scores, SGA, and LGA in this large sample of pregnancies across the United States. We also evaluated effect modification by region, fetal sex, maternal race, and neighborhood poverty to identify potentially vulnerable subgroups.

## Methods

### Study population and outcomes

The study population was drawn from the ECHO Cohort; detailed cohort characteristics and data harmonization procedures are described elsewhere.^44,45^ Following the inclusion and exclusion criteria described previously (Figure S1, https://links.lww.com/EE/A468),^43^ we included consented ECHO participants with live singleton births and dates of conception from 1 January 2006 to 20 March 2020 and available pregnancy residential history. We excluded participants residing outside the contiguous United States. We also excluded participants from cohort sites that recruited based on low gestational age or birth weight, sites with fewer than 100 eligible participants, and sites with >25% missing data in primary covariates. Study activities were approved by local institutional review boards (IRBs) and/or the single ECHO IRB, and participants provided prior written informed consent for future use of data for ECHO-wide research.

Our primary outcome of interest was birth weight at term (weight in grams at birth among infants born at least 37 weeks of gestation). Only term births (at least 37 weeks of gestation) were included to ensure that lower birth weight among preterm infants would not drive effect estimates.^43^ Wildfire smoke exposure may also contribute to associations with both low and high birth weight that are obscured in an analysis of linear birth weight at term.^29,33^ We therefore included the additional secondary outcomes of sex-adjusted BWGA *z*-scores, SGA (weight <10th percentile for sex-specific singleton births), and LGA (weight >90th percentile for sex-specific singleton births), based on the Aris et al^46^ US reference population.

### Exposure

We assigned exposure using a previously described machine learning model that estimates daily wildfire PM_2.5_ concentrations at a 10-km spatial resolution in the contiguous United States from 2006 to 2020.^47^ As described in Childs et al,^47^ days with wildfire smoke and related wildfire PM_2.5_ levels at ground-level U.S. EPA monitors were identified using satellite imagery and Hybrid Single-Particle Lagrangian Integrated Trajectory model (HYSPLIT) simulated air trajectories originating from fires. Spatiotemporal inputs including meteorology, smoke and fire variables, aerosol measurements, and land cover were used to train a machine learning model predicting wildfire PM_2.5_ at a 10 km spatial resolution using spatiotemporal input data, ultimately generating population-weighted averages for each census tract. This model demonstrated strong performance on independent datasets, with an out-of-sample *R*^2^ ranging from 0.67 to 0.70 across the full spectrum of wildfire PM_2.5_ exposure levels. However, model performance did vary regionally, with the highest *R*^2^ in the West and Northeast.

We linked daily estimates to study participants based on the date and residential census tract during pregnancy. Pregnancy exposure for the outcome of birth weight at term was calculated from 0 to 36 weeks of gestation; pregnancy exposure for secondary outcomes was calculated from 0 to 31 weeks. We calculated the average (arithmetic mean) concentration of daily wildfire PM_2.5_, the number of “smoke days” with measurable wildfire PM_2.5_ (>0 µg/m^3^), and the number of smoke days above predefined concentration thresholds (≥2.5, ≥5, or ≥10 µg/m^3^) based on the distribution of wildfire PM_2.5_ on smoke days.^43^ As a secondary analysis to capture multiday smoke episodes, we quantified the number of “smoke waves” defined as consecutive smoke days (2, 3, or 4+) at the same thresholds. To enable analysis of sensitive exposure windows across pregnancy, we evaluated the number of overall smoke days (>0 µg/m^3^) in each week of pregnancy, as well as average wildfire PM_2.5_ and smoke days in trimester 1 (week 0–13), trimester 2 (week 14–26), and trimester 3 (the last 4 weeks of pregnancy, to avoid correlation between exposure metrics and length of gestation).

### Statistical analysis

For descriptive statistics, we calculated the mean pregnancy wildfire PM_2.5_ across pregnancy (conception through delivery) in the study population and by cohort study site. We also calculated the percentage of participants experiencing smoke days of each intensity in the study population.

Associations between wildfire PM_2.5_ exposure and the study outcomes were determined using mixed-effects linear regression (birth weight at term and BWGA *z*-scores) or mixed-effects logistic regression (SGA and LGA). We used multiple imputation by chained equations within each cohort study site via the “mice” and “miceadds” packages in R to address missing covariate data, following the analytic framework described previously.^43^ All models incorporated study site random intercepts to account for the correlation of baseline outcome risks among participants within the same site. Primary models adjusted for key confounders and precision variables including demographic factors (fetal sex, maternal age at delivery, education, and self-reported race and Hispanic ethnicity), residential socioeconomic context (census-tract poverty rate), and temporal trends (conception season and birth year). Spatial splines with 10 degrees of freedom were included to account for unmeasured spatial confounding at the regional scale (Table S1, https://links.lww.com/EE/A468).^48^ Extended models incorporated additional covariates including parity, maternal pre-pregnancy body mass index, self-reported pregnancy tobacco use, and self-reported pregnancy alcohol consumption. Sites with over 50% missingness in any covariate were excluded from extended models.

We performed various sensitivity analyses. Models with the same adjustments as the primary model were also fit using the restricted sample (i.e., the sample used for the extended model) in order to allow direct comparison between adjustment approaches in the same population. To explore the impact of design choices to account for seasonal confounding, we adjusted for conception month and date of conception splines (instead of season of conception) in separate models. We additionally adjusted for pregnancy-average overall ambient PM_2.5_ and ambient temperature to evaluate the potential role of co-exposures.^49,50^ We also conducted complete case analysis instead of multiple imputation.

We conducted two sensitivity analyses to further evaluate the impact of modeling choices related to geographic variation and site structure. First, we fit models including both site-specific random intercepts and random slopes for wildfire smoke exposure. These models allow for more complex correlation among participants in the same site than the random intercept model by incorporating variability in the exposure-outcome association between sites. Second, we fit models including site random intercepts but excluding spatial spline terms; these models account for correlation of baseline outcome risks among participants within the same site but do not adjust for unmeasured spatial confounding. Third, we fit models without adjustment for site but including spatial spline terms.

Finally, we repeated the analysis of secondary outcomes (BWGA *z*-scores, SGA, LGA) after excluding births <32 weeks gestational age at delivery to allow a consistent pregnancy exposure window (0–31 weeks) to avoid potential bias induced by correlation between gestational age at delivery and cumulative exposure metrics, as well as between gestational age and likelihood of SGA/LGA.

To evaluate sensitive exposure windows across gestation, we used two approaches: distributed lag models (DLMs) and trimester-specific associations. DLMs included weekly measures of smoke days from 0 to 36 weeks of gestation for the outcome of birth weight at term, and from 0 to 31 weeks for the outcomes of BWGA *z*-scores, SGA, and LGA (to ensure that births ≥32 weeks of gestational age would be included in the analysis). Linear DLMs were implemented with the “dlnm” package in R. Models were implemented for each of the 10 multiple imputation by chained equations–imputed analytic datasets, and pooled effect estimates and standard errors were calculated by Rubin’s rules. Trimester-specific associations were evaluated for the exposures of wildfire PM_2.5_ and overall smoke days in models mutually adjusted for all trimesters with adjustment for primary covariates. We performed post hoc sensitivity analyses to investigate the potential for residual seasonal confounding by repeating DLMs with more flexible adjustment for seasonality via (1) conception month and (2) date of conception splines (instead of season of conception), similar to sensitivity analyses described earlier.

To identify potentially vulnerable subgroups, we evaluated effect modification by the four US Census regions, fetal sex, maternal self-reported race, and census-tract neighborhood poverty rate. We included interaction terms between modifiers of interest and wildfire metrics (mean pregnancy PM_2.5_ and smoke days) and conducted stratified models, adjusted for primary covariates.

## Results

### Study population characteristics

There were 22,656 singleton pregnancies in ECHO from 47 cohort sites with complete exposure and outcome data for consideration (Figure S1, https://links.lww.com/EE/A468). We excluded 1490 births from six sites that recruited based on gestational age or birth weight, 691 births from two sites with high missingness of primary covariates, and 441 births from 10 sites with insufficient eligible participants. This yielded a study population of 20,034 participants from 30 cohort sites with prenatal residential history in all 48 contiguous US states (Table S1, https://links.lww.com/EE/A468). N = 21 births were missing information on secondary outcomes.

Around half (51.2%) of infants were male (Table 1). The median maternal age at delivery was 31 years. Maternal race and ethnicity distributions were as follows: 62.3% White, 12.7% Black, 6.7% Asian, Native Hawaiian, or Pacific Islander, 2.0% American Indian or Alaska Native, and 22.0% Hispanic. The “restricted” analytic sample excluded an additional 3,807 participants from study sites with >50% missingness of extended model covariates, for a restricted sample of N = 16,227 dyads from 18 sites (Figure S1, https://links.lww.com/EE/A468) with similar population characteristics (Table S2, https://links.lww.com/EE/A468).

**Table 1.** Study population characteristics

	Overall (N = 20,034)
Fetal sex	
Male	10,248 (51.2%)
Female	9,779 (48.8%)
Missing	7 (0.0%)
Maternal age at delivery	
Mean (SD)	30.6 (5.58)
Median [Min, Max]	31.0 [14.0, 58.0]
Missing	53 (0.3%)
Maternal race	
White	12,482 (62.3%)
Black	2,553 (12.7%)
Asian, Native Hawaiian, or other Pacific Islander	1,342 (6.7%)
American Indian or Alaska Native	400 (2.0%)
More than one race or Other Race	1,876 (9.4%)
Missing	1,381 (6.9%)
Maternal ethnicity	
Hispanic	4,417 (22.0%)
Non-Hispanic	15,191 (75.8%)
Missing	426 (2.1%)
Maternal educational level	
High school degree or less	4,372 (21.8%)
Some college, Associate’s degree, or trade school	3,860 (19.3%)
Bachelor’s degree	4,743 (23.7%)
Postgraduate degree	4,314 (21.5%)
Missing	2,745 (13.7%)
Parity	
1	6,967 (34.8%)
2	6,064 (30.3%)
3 or more	4,151 (20.7%)
Missing	2,852 (14.2%)
Pregnancy tobacco use	
Yes	1,418 (7.1%)
No	16,173 (80.7%)
Missing	2,443 (12.2%)
Pregnancy alcohol consumption	
Yes	3,233 (16.1%)
No	13,117 (65.5%)
Missing	3,684 (18.4%)
Census region	
Midwest	3,570 (17.8%)
Northeast	6,379 (31.8%)
South	4,278 (21.4%)
West	5,807 (29.0%)
Conception season	
Autumn	5,337 (26.6%)
Spring	4,693 (23.4%)
Summer	5,096 (25.4%)
Winter	4,908 (24.5%)
Birth year	
2006–2009	1,569 (7.8%)
2010–2013	6,111 (30.5%)
2014–2017	7,099 (35.4%)
2018–2021	5,255 (26.2%)

There were 18,347 full-term pregnancies in the study population (91.6%). Among these, the average birth weight at term was 3411 g (standard deviation [SD]: 459 g) (Table 2). The range of birth weight at term was from 1470 to 5925 g. Among 20,013 births with available secondary outcomes, the mean *z*-score was 0.04 (SD: 1.07), with a minimum of −6.58 and a maximum of 5.14. The prevalence of SGA was 10.5%, and the prevalence of LGA was 11.6%.

**Table 2.** Distribution of birth weight outcomes in the study population

Outcome	Mean	N	SD	Median	IQR	Minimum	Maximum
Birth weight at term (g)	18,347	3,411	459	3,402	601	1,470	5,925
BWGA z-score	20,013	0.04	1.07	0.01	1.39	−6.58	5.14
	**N**	**Frequency**	**Percent**				
SGA	20,013						
Yes		2,095	10.5%				
No		17,918	89.5%				
LGA	20,013						
Yes		2,327	11.6%				
No		17,686	88.4%				

### Pregnancy wildfire PM_2.5_ exposure

Prenatal wildfire PM_2.5_ exposure was generally low but nearly ubiquitous, with average concentrations of 0.36 μg/m^3^ (SD: 0.46). The average wildfire PM_2.5_ concentrations in cohort study sites ranged from 0.14 to 0.95 µg/m^3^ (Table S1, https://links.lww.com/EE/A468). Over 99% of participants experienced at least one smoke day of any concentration during pregnancy; 96.5% experienced a smoke day of ≥2.5 µg/m^3^, 84.7% experienced a smoke day of ≥5 µg/m^3^, and 48.7% experienced a smoke day of ≥10 µg/m^3^. Prior work in the same study population has shown that average wildfire PM_2.5_ and number of smoke days across pregnancy were highest in the West and Midwest relative to the South and Northeast US census regions.^43^ Wildfire PM_2.5_ exposure was highest among participants identifying as Asian, Native Hawaiian, or Other Pacific Islander or American Indian or Alaska Native relative to White and Black participants.^43^

### Analytic results

#### Primary results

There was no association between average wildfire PM_2.5_ or smoke days of varying intensities and the primary outcome of birth weight at term (Table 3). There were also no consistent associations observed for BWGA *z*-scores and LGA. While estimates were in the positive direction for SGA, none reached statistical significance. There were no clear associations between smoke waves of varying intensity and duration and any of the primary or secondary outcomes (Table S3, https://links.lww.com/EE/A468).

**Table 3.** Association between wildfire PM_2.5_ metrics and birth weight outcomes determined using mixed-effects linear and logistic regression adjusted for maternal age, race, Hispanic ethnicity, and education; child sex; neighborhood poverty rate; season of conception, year of birth splines (4 df), spatial splines (10 df), and cohort random intercept

Exposure	Coefficient and 95% CI		OR and 95% CI	
	Term birth weight (g)	BWGA *z*-scores	SGA	LGA
Mean wildfire PM_2.5_	7.4 (−8.5, 23.4)	0.005 (−0.031, 0.041)	1.051 (0.937, 1.179)	1.055 (0.950, 1.173)
Smoke days				
≥0 µg/m^3^	−0.2 (−0.8, 0.4)	−0.001 (−0.002, 0.000)	1.002 (0.997, 1.006)	0.999 (0.995, 1.003)
≥0 µg/m^3^	−0.1 (−0.9, 0.7)	−0.001 (−0.003, 0.001)	1.002 (0.996, 1.008)	1.000 (0.995, 1.005)
≥0 µg/m^3^	−0.3 (−1.5, 1.0)	−0.002 (−0.004, 0.001)	1.005 (0.996, 1.013)	1.000 (0.992, 1.008)
≥0 µg/m^3^	0.0 (−2.5, 2.4)	−0.001 (−0.007, 0.004)	1.007 (0.991, 1.024)	1.008 (0.992, 1.024)

Associations are interpreted per 1 µg/m^3^ change in mean wildfire PM_2.5_ or with a 1-day increase in smoke days during pregnancy.

Findings for birth weight at term, BWGA *z*-scores, and SGA were similar with adjustment for extended covariates and with primary adjustment in the restricted sample (Table S4, https://links.lww.com/EE/A468). Associations between average wildfire PM_2.5_ and LGA were strengthened slightly with more comprehensive adjustment (odds ratio [OR] = 1.076 per µg/m^3^, 95% confidence interval [CI]: 0.966, 1.199). Compared with the estimates of primary models, associations were largely unchanged in sensitivity analyses including adjustment for ambient temperature, alternate model specifications, and more flexible adjustment for conception season (Table S5, https://links.lww.com/EE/A468). Adjustment for total ambient PM_2.5_ somewhat attenuated associations between average wildfire PM_2.5_ and SGA and LGA.

#### Role of exposure timing

**Figure 1.**
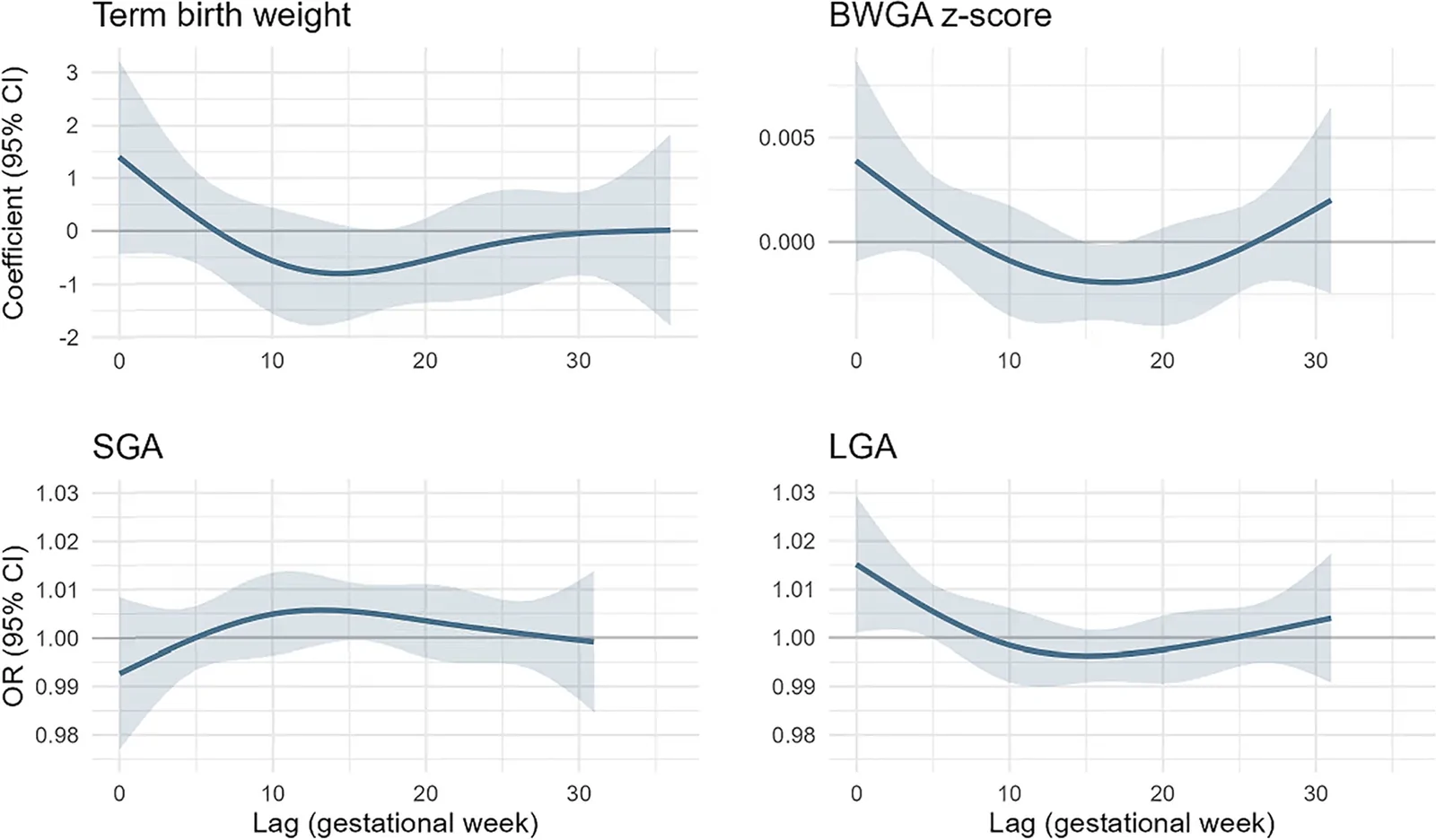
Lagged associations between wildfire smoke days (≥0 µg/m^3^) and outcomes determined by DLMs adjusted for maternal age, race, Hispanic ethnicity, and education; child sex; neighborhood poverty rate; season of conception, year of birth splines (4 df), spatial splines (10 df), and cohort random intercept.

DLMs identified small adverse associations between mid-pregnancy smoke days and outcomes related to fetal growth restriction. A small reduction in birth weight at term was most precise in week 14 (β = −7.4 g per smoke day, 95% CI: −1.5, 0.02) (Figure 1). The lagged association between smoke days and BWGA *z*-scores followed a U-shaped distribution, with the strongest associations in mid-pregnancy (β = −0.0019 per smoke day in gestational week 17, 95% CI: −0.0038, −0.00008). Lagged associations with SGA were null, but with a small increase in odds around gestational week 13 (OR = 1.006, 95% CI: 0.998, 1.013). In contrast, smoke days in early pregnancy were associated with a modest increase in odds of LGA (OR = 1.015 per smoke day in gestational week 0, 95% CI: 1.001, 1.029).

In post hoc analyses exploring more flexible seasonal adjustment, the U-shaped distribution for birth weight at term and BWGA *z*-scores was attenuated when adjusting for month of conception or date of conception splines; mid-pregnancy associations for BWGA *z*-scores became nonstatistically significant (Figure S2, https://links.lww.com/EE/A468). Mid-pregnancy associations with SGA were also attenuated. However, the early-pregnancy association between smoke days and LGA was strengthened with adjustment for date of conception splines (OR = 1.021 per smoke day in gestational week 0, 95% CI: 1.004, 1.038).

In analyses of trimester-specific associations, the primary wildfire exposure metrics (average wildfire PM2.5 and smoke days) were not associated with birth weight at term, BWGA *z*-scores, or SGA in any trimester (Table S6 https://links.lww.com/EE/A468). However, there was a small association between first-trimester average wildfire PM_2.5_ and higher odds of LGA (OR = 1.066, 95% CI: 0.999, 1.138).

#### Effect modification

We did not identify consistent evidence of effect modification by region, infant sex, maternal self-identified race, or poverty tertile (Figure S3, https://links.lww.com/EE/A468) in the association between average wildfire PM_2.5_ or smoke days and the primary outcome of birth weight at term. Stratified associations between potential modifiers and wildfire PM_2.5_ and smoke days were null, and all interaction *P* values were greater than 0.20.

## Discussion

We estimated associations between wildfire PM_2.5_ exposure during pregnancy and birth weight-related outcomes in the prospective, nationwide U.S. ECHO Cohort. Our study population included over 20,000 participants from 30 pregnancy and pediatric cohort sites, with participants residing in all 48 contiguous states. We did not identify associations between pregnancy-average exposure and the primary outcome of birth weight at term or secondary outcomes of BWGA *z*-scores, SGA, and LGA. DLMs identified marginal adverse associations between mid-pregnancy exposure to smoke days and birth weight at term BWGA, *z*-scores, and SGA, but these were attenuated with more flexible seasonal adjustment and not observed in trimester-specific analyses. We found a small association between early-pregnancy smoke days and LGA, which was also reflected in trimester-specific analyses. We did not find evidence of effect modification by any of the factors examined.

Pregnancy exposure to total ambient PM_2.5_ has been robustly associated with lower birth weight and related outcomes,^21,22,51^ though restriction to term births reduced the effect size in meta-analyses.^52^ Emerging evidence also points to an association between wildfire PM_2.5_ and decreased birth weight, though findings have been variable. A recent meta-analysis found that eleven of thirteen studies observed lower birth weight with increasing wildfire smoke exposure, and nine of ten studies reported higher odds of low birth weight (<2,500 g).^29^

In contrast, our study identified largely null results for outcomes related to fetal growth restriction. One important reason for this distinction may be that our analysis either restricted to term births or used gestational age–specific metrics of BWGA *z*-scores, SGA, and LGA. Prior studies, including in the same ECHO population, have identified associations between prenatal wildfire smoke exposure and preterm birth.^29,37,39,43^ Therefore, links between wildfire smoke and outcomes of low birth weight and continuous birth weight may be driven at least in part by mechanisms related to reduced gestational age. Indeed, the limited prior research on wildfire smoke and birth weight among term births has found much less consistent evidence of adverse effects relative to those that do not condition on gestational age at delivery. In one study restricted to term births, an increase in the number of wildfires from 2001 to 2018 in Brazil municipalities was associated with increased risk of low birth weight in only two of five regions; inverse associations were identified in one region.^53^ Zhang et al^37^ identified significant associations between an IQR (1.25 µg/m^3^) increase in wildfire PM_2.5_ in New South Wales and term low birth weight (hazard ratio: 1.036, 95% CI: 1.014, 1.058), while Jiang et al^39^ observed null associations for term low birth weight in eight US states.

There could also be other reasons for the observed null associations, including limited statistical power to evaluate the relatively rare outcomes of SGA and LGA, particularly compared with prior studies that have used large sample sizes of administrative records. Reduced precision from exposure measurement error could also contribute to null findings, as discussed in detail later. Heterogeneity in findings between prior studies of wildfire smoke and adverse birth outcomes and the present study may also stem from differences in exposure assessment methods, study design, adjustment for confounding, geographic settings of focus, and outcome assessment.^7,29^ In contrast to the present study, all prior research relied on administrative birth records to determine outcomes, while we use data from prospective birth cohorts with available address history and robust individual-level covariate data.^29^

Our analytic approach aimed to balance control of site-level and spatial confounding with preservation of meaningful geographic variation in wildfire smoke exposure. Models incorporating site random intercepts account for clustering and differences in baseline outcome risk across sites, while partially attenuating the influence of between-site exposure differences on the estimated association. Spatial spline terms further adjusted for unmeasured confounding related to broad regional gradients in exposure and outcome risk. Sensitivity analyses allowing site-specific exposure effects (random slopes), excluding spatial splines, and excluding site adjustment yielded results consistent with the primary analysis, suggesting that our findings are robust to alternative specifications of site and spatial structure.

Our analysis did not find consistent evidence for an association between entire-pregnancy wildfire smoke exposure and outcomes related to fetal growth restriction, including birth weight at term, BWGA *z*-scores, and SGA. DLMs identified some marginal associations between exposures in mid-pregnancy and these outcomes. In contrast, five recent studies on wildfire smoke exposure and birth weight observed the strongest associations for exposure in the first trimester of pregnancy.^29–31,33,37,38^ The U-shaped relationship between smoke day exposure and birth weight outcomes across pregnancy in our analysis lacks a straightforward biological explanation. We explored the potential for residual seasonal confounding in post hoc analyses with more flexible seasonal adjustment, given notable trends in the seasonality of both wildfire exposure and birth weight outcomes.^54,55^ These analyses resulted in a more even distribution and attenuation of associations for most outcomes. This suggests that adjustment for conception season as a four-level categorical variable may not be sufficient to counter confounding by seasonality of exposures and outcomes in lagged associations.

Effect modification analyses did not reveal significant differences in associations between wildfire smoke exposure and birth weight at term across strata defined by region, fetal sex, maternal race, or neighborhood poverty. A prior study likewise did not find evidence of effect modification by maternal race/ethnicity or markers of socioeconomic status (education and insurance coverage) in the association between wildfire PM_2.5_ and term low birth weight in the Southwestern United States.^39^ One study in Australia found an association between exposure to wildfires during pregnancy and risk of LGA only among male infants, but not female infants.^34^ Another study found no evidence of sex-specific differences in the associations between average wildfire PM_2.5_ or smoke days >5 µg/m^3^ and BWGA *z*-scores.^33^ Our analysis used neighborhood-level poverty rate and self-reported maternal race/ethnicity as proxies for structural barriers, yet these metrics may not sufficiently capture underlying risk or resilience factors potentially modifying associations between wildfire exposure and pregnancy outcomes.

Our analysis provides some limited evidence for an association between early-pregnancy wildfire smoke exposure and LGA. LGA infants have an increased risk of birth trauma and other neonatal complications and morbidity, as well as an increased risk of long-term health consequences including metabolic disorders and cardiovascular disease.^28,56^ Prior studies have also identified associations between wildfire smoke^33–35^ or total ambient PM_2.5_^26^ and measures of fetal overgrowth including LGA and macrosomia (birth weight >4,000 g), with mixed findings related to critical windows of exposure. These associations may be driven by indirect or direct pathways including maternal metabolic disorders, alterations in nutrient transport across the placenta, epigenetic modification, inflammation, or oxidative stress.^26,57^ Some ECHO cohort sites collected information on maternal metabolic disorders during pregnancy including gestational diabetes mellitus; future research evaluating these outcomes in direct association with prenatal wildfire smoke and as potential mediators of fetal overgrowth would clarify the potential biological mechanisms and critical windows of exposure.

This study has limitations. Evaluating associations between exposure and birth weight only among term births may introduce bias by conditioning on a collider (gestational age). The outcome of SGA also has been criticized as a poor measure of fetal growth restriction and a predictor of later life adverse health outcomes.^58,59^ To mitigate these issues, we also evaluated the related outcome of BWGA *z*-scores. Our exposure estimates were derived from a statistical model with known spatial variability in performance, as described earlier. Both classical and Berkson-type exposure misclassification are possible. The relatively coarse spatial resolution of the model (census tract), in particular, may lead to Berkson-type misclassification; while this type of misclassification is expected to result in reduced precision rather than bias, it may contribute to observed null effects.^60^ Because plume detection does not differentiate wildfire smoke from other biomass-burning sources, misclassification of agricultural or refuse burning as non-wildfire smoke is possible. Nonetheless, the model was chosen for its strong cross-validated performance, nationwide coverage, and prior validation against independent estimates.^61^ Finally, because we examined numerous exposure–response relationships, the likelihood of type 1 error was elevated. Rather than interpreting isolated significant findings, we emphasized consistent patterns across related exposure metrics.

This study has several important strengths that enhance the validity and generalizability of findings. We evaluated multiple complementary indicators of fetal growth, enabling assessment of both subtle distributional shifts and clinically defined extremes. We also extended the prior literature by considering pathways related to both fetal growth restriction and fetal overgrowth. Our analysis also evaluated the role of smoke days at multiple concentration thresholds chosen to reflect a range of exposure intensities relevant to public health guidance. The application of DLMs allowed us to examine potential sensitive windows of fetal growth, an approach rarely applied to birth weight outcomes in wildfire smoke research. The use of data from pooled ECHO sites is a strength of our study, allowing us to leverage a geographically, socioeconomically, and demographically diverse study population across the contiguous United States. Our study period spanned approximately 14 years, providing substantial temporal exposure variability. Ours is also among the first analyses of wildfire smoke and birth weight in a longitudinal birth cohort. We therefore were able to access information not typically available or complete in administrative records, including pregnancy tobacco use, pregnancy alcohol consumption, pre-pregnancy BMI, and maternal residential history across pregnancy.

In this nationwide longitudinal cohort, we observed limited evidence that prenatal exposure to wildfire PM_2_._5_ was associated with fetal growth restriction. Across multiple exposure metrics and growth indicators, associations with birth weight, BWGA *z*-scores, and SGA were generally null. Modest signals emerged for increased risk of LGA with early-pregnancy smoke. These findings suggest that wildfire smoke may influence fetal growth through pathways that differ from those underlying shortened gestation and highlight the importance of considering fetal overgrowth alongside growth restriction in studies of wildfire smoke and pregnancy outcomes. As wildfire activity continues to intensify and expand geographically, further research is needed to clarify biological mechanisms, identify susceptible windows of exposure, and determine whether high-intensity or prolonged smoke events have clinically meaningful impacts on fetal growth and other early life health outcomes. Such evidence will be critical for informing guidance aimed at protecting maternal and infant health during wildfire smoke episodes.

## Conflict of interest statement

A.E.H. was a member of a data safety and monitoring board for a treatment trial of blood pressure management at the University of Pittsburgh. A.R.S. consulted for UNICEF on an analysis of child exposure to pesticides. C.J.K. received an honorarium from Kaiser Permanente to present for pediatricians on wildfire smoke and child health, and received an honorarium and travel support from California Chapter 2 of the American Academy of Pediatrics to present on provider medical education related to air pollution and child health. C.T.M. was chair of a data safety and monitoring board for a US National Institutes of Health (NIH) study, Safety of Sildenafil in Premature Infants with Severe Bronchopulmonary Dysplasia (SILDI-SAFE), and member of a data safety and monitoring board for the Pragmatic Research on Diuretic Management in Early BPD (PRIMED) Study. C.T.M. also received annual fees and royalties as a peer reviewer for UpToDate, and honoraria for lectures including an Endowed Lectureship at the University of Illinois in March 2024, the Vermont Oxford Hot Topics Meeting in December 2024, and the Mednax Center for Research, Education, Quality and Safety Annual Conference for Neonatology in February 2025. DH was a member of an advisory committee for the Targeting Environmental Neurodevelopmental Risks Project. J.A. served on the Avera Health Institutional Review Board. R.J.S. has received funding support from the Bia-Echo Foundation. R.J.S. also consults for Linus Technology and consulted for the Beasley Law Firm until March 2023. R.J.S. also received travel support to serve on the Observational Study Monitoring Board for the Healthy Brain and Child Development Study, to present at the BC Lung Foundation Air Quality & Health Workshop 2025 (April 2025), to present at the Japan Society of Endocrine Disruptors Research Meeting (December 2024), to present at the Society for Birth Defects Research and Prevention 64th Annual Meeting (June 2024), and to present at the 35th Annual Meeting of the Organization of Teratology Information Specialists (June 2023). All other authors declare no competing interests.

## Acknowledgments

The authors wish to thank our ECHO Colleagues; the medical, nursing, and program staff; and the children and families participating in the ECHO Cohort.

## Supplementary Material


