# Supplementary material for: Prenatal exposure to wildfire fine particulate matter and fetal growth outcomes in the United States: Findings from the ECHO Cohort
Source: Environ Epidemiol. 2026 Sep 9;10(5):e526. doi: 10.1097/EE9.0000000000000526 (PMC13561280; doi:10.1097/EE9.0000000000000526)
Supplement: Supplementary file 1 [file ee9-10-e526-s001.pdf]

Exposure to wildfire-derived fine particulate matter during pregnancy and birth weight outcomes in the U.S. ECHO Cohort:

Supplemental materials

Contents

Table S1. Cohort study site characteristics 2

Table S2. Characteristics of restricted study population 3

Table S3. Association between smoke wave metrics and study outcomes 4

Table S4. Associations between exposures and outcomes in the restricted population with primary and extended model adjustment 5

Table S5. Results of sensitivity analyses. 6

Table S6. Results of trimester-specific analyses 7

Table S7. ECHO Cohort Consortium Collaborators 8

Figure S1. Inclusion flowchart. Reproduced with permission from Sherris et al. (2025) 15

Figure S2. Results of distributed lag models with more flexible seasonal adjustment 16

Figure S3. Effect modification in the relationship between wildfire $PM_{2.5}$ exposure and birth weight at term (g). 17

Table S1. Cohort study site characteristics

Cohort ID	Cohort Name	N	Average WF $PM_{2.5}$ (μg/m3)	Birth years	Recruitment site locations
10402	43rd Multicenter Airway Research Collaboration (MARC-43)	275	0.14	2013-2017	Boston, MA; Philadelphia, PA; Louisville, KY; Phoenix, AZ
10601	Healthy Start	841	0.41	2010-2014	Aurora, CO
10703	MINNIE	402	0.26	2018-2020	Denver, CO
10801	Boricua Youth Study (BYS)	129	0.23	2010-2020	New York, NY
10901	Atlanta ECHO Cohort of Emory University	287	0.20	2014-2020	Atlanta, GA
11001	Safe Passage Study (PASS)	2,169	0.55	2008-2020	Rapid City, SD; Sioux Fans, SD
11201	Pregnancy Environment and Lifestyle study (PETALS)	1,115	0.75	2014-2019	Richmond, CA; Union City, CA; San Leandro, CA; San Jose, CA
11202	Kaiser Permanente Research Bank (KPRB)	641	0.45	2014-2018	California (Bay Area and Central Valley)
11401	Maternal and Development Risks from Environmental and social stressors (MADRES)	383	0.27	2016-2020	Los Angeles, CA
11601	Revisiting CHildhood Autism Risks from Genes and the Environment Study (ReCHARGE)	337	0.48	2006-2014	Sacramento, CA; Los Angeles, CA
11701	Pittsburgh Gins study (PGS)	156	0.23	2012-2020	Pittsburgh, PA
11801	New Hampshire Birth Cohort study (NHBCS)	2,006	0.21	2009-2020	Lebanon, NH; Concord, NH; Warner, NH
11901	Conditions Affecting Neurocognitive Development and Learning in Early Childh00d (CANDLE)	815	0.39	2007-2011	Memphis, TN
11903	The Global Alliance to Prevent Prematurity and stillbirth (GAPPS)	503	0.47	2011-2017	Seattle, WA; Yakima, WA
12100	Early Growth and Development Study Pediatric Cohort	245	0.39	2006-2015	Various locations (CA, VA, NJ, TX, OR, WA, FL, UT, IL, MD, PA)
12301	Vitamin D Antenatal Asthma Reduction TOA (VDAART)	393	0.30	2010-2012	Boston, MA; San Diego, CA, St. Louis, MO
12401	Vitamin C to Decrease Effects of Smoking in Pregnancy on Infant Lung Function (VCSIP)	194	0.31	2013-2016	Indianapolis, IN; Portland, OR; Vancouver, WA
12402	In-Utero Smoke, Vitamin C, and Newborn Lung Function	115	0.21	2007-2011	Portland, OR; Vancouver, WA
12514	Infant Brain Imaging Study (IBIS)	122	0.28	2007-2017	Philadelphia, PA; Chapel Hill, NC; Seattle, WA
12601	Rochester	179	0.33	2016-2020	Rochester, NY; Pittsburgh, PA
12602	Magee	161	0.31	2017-2020	Rochester, NY; Pittsburgh, PA
12901	Archive for Research in Child Health (ARCH)	463	0.37	2008-2017	Lansing, MI
12902	Michigan Archive for Research in Child Health (MARCH)	582	0.45	2018-2020	Michigan (statewide)
13102	Chemicals in our Bodies (CIOB)	454	0.95	2014-2020	San Francisco, CA
13200	Utah Children's Project	690	0.26	2006-2018	Salt Lake City, UT; Logan, UT
13301	The NYU Children's Health and Environment Study (NYU CHES)	1,877	0.24	2016-2020	New York, NY
13501	Asthma Coalition on Community, Environment & Social Stress (ACCESS)	163	0.26	2006-2008	Boston, MA; Roxbury, MA; Brockton, MA; Dorchester, MA
13502	PRogramming of Intergenerational Stress Mechanisms (PRISM)	864	0.26	2011-2020	East Boston, MA; Boston, MA; New York, NY
13503	First 1000 Days	2,959	0.23	2011-2020	Falls Church, VA
20101	The Infant Development and the Environment Study (TIDES)	514	0.30	2011-2013	Seattle, WA, San Francisco, CA; Minneapolis, MN; Rochester, NY

Table S2. Characteristics of restricted study population

	Overall (N=16227)
**Child sex**	
Male	8296 (51.1%)
Female	7924 (48.8%)
Missing	7 (0.0%)
**Age of the pregnant individual**	
Mean (SD)	30.9 (5.51)
Missing	8 (0.0%)
**Race of the pregnant individual**	
White	10567 (65.1%)
Black	1584 (9.8%)
Asian, Native Hawaiian, or other Pacific Islander	1209 (7.5%)
American Indian or Alaska Native	371 (2.3%)
More than one race or Other Race	1356 (8.4%)
Missing	1140 (7.0%)
**Ethnicity of the pregnant individual**	
Hispanic	3537 (21.8%)
Non-Hispanic	12356 (76.1%)
Missing	334 (2.1%)
**Educational level of the pregnant individual**	
High school degree or less	3383 (20.8%)
Some college, Associate's degree, or Trade school	3033 (18.7%)
Bachelor's degree	4083 (25.2%)
Postgraduate degree	3622 (22.3%)
Missing	2106 (13.0%)
**Parity**	
1	6201 (38.2%)
2	5423 (33.4%)
3 or more	3601 (22.2%)
Missing	1002 (6.2%)
**Pregnancy tobacco use**	
Yes	1163 (7.2%)
No	14115 (87.0%)
Missing	949 (5.8%)
**Pregnancy alcohol consumption**	
Yes	2818 (17.4%)
No	11410 (70.3%)
Missing	1999 (12.3%)
**Census region**	
Midwest	2838 (17.5%)
Northeast	4004 (24.7%)
South	4159 (25.6%)
West	5226 (32.2%)
**Conception season**	
Autumn	4311 (26.6%)
Spring	3856 (23.8%)
Summer	4210 (25.9%)
Winter	3850 (23.7%)
**Birth year**	
2006-2009	1208 (7.4%)
2010-2013	4582 (28.2%)
2014-2017	6241 (38.5%)
2018-2021	4196 (25.9%)

Table S3. Association between smoke wave metrics and study outcomes

Smoke wave intensity and duration	Coefficient and 95% CI		OR and 95% CI	
	Term birth weight (g)	BWGA z-scores	SGA	LGA
≥ 2.5 μg/m^3				
2 days	-1.6 (-5.4, 2.1)	-0.006 (-0.014, 0.002)	1.015 (0.988, 1.043)	0.991 (0.968, 1.015)
3 days	-2.9 (-8.5, 2.8)	-0.009 (-0.022, 0.003)	1.025 (0.984, 1.067)	0.993 (0.957, 1.030)
4+ days	-2.9 (-10.8, 4.9)	-0.016 (-0.034, 0.001)	1.040 (0.984, 1.099)	0.992 (0.943, 1.044)
≥ 5.0 μg/m^3				
2 days	-2.4 (-7.7, 2.9)	-0.007 (-0.019, 0.004)	1.026 (0.988, 1.065)	0.986 (0.953, 1.020)
3 days	-3.7 (-11.8, 4.5)	-0.014 (-0.032, 0.003)	1.042 (0.983, 1.104)	0.985 (0.935, 1.037)
4+ days	-2.9 (-14.7, 8.9)	-0.018 (-0.044, 0.008)	1.016 (0.936, 1.103)	1.013 (0.939, 1.094)
≥ 10 μg/m^3				
2 days	-4.2 (-14.2, 5.8)	-0.007 (-0.030, 0.015)	1.026 (0.957, 1.099)	1.021 (0.954, 1.093)
3 days	2.9 (-13.8, 19.6)	-0.001 (-0.037, 0.036)	1.026 (0.915, 1.151)	1.087 (0.975, 1.212)
4+ days	-1.1 (-23.4, 21.2)	-0.022 (-0.071, 0.027)	1.079 (0.926, 1.258)	1.072 (0.930, 1.236)

Table S4. Associations between exposures and outcomes in the restricted population with primary and extended model adjustment (N=16,227). Estimates represent coefficient and 95% CI for term birth weight and BWGA z-scores and OR and 95% CI for SGA and LGA.

Outcome	Exposure	Primary Model	Extended Model
Term birth weight	Average wildfire $PM_{2.5}$	7.4 (-8.6, 23.4)	10.8 (-5.1, 26.7)
	Smoke days (overall)	-0.2 (-0.8, 0.4)	-0.2 (-0.8, 0.4)
	Smoke days (≥ 2.5 μg/m^3)	-0.1 (-0.9, 0.7)	-0.0 (-0.9, 0.7)
	Smoke days (≥5 μg/m^3)	-0.3 (-1.5, 1.0)	-0.2 (-1.5, 1.0)
	Smoke days (≥10 μg/m^3)	-0.0 (-2.5, 2.4)	0.4 (-2.0, 2.8)
BWGA z-scores	Average wildfire $PM_{2.5}$	0.005 (-0.032, 0.041)	0.011 (-0.025, 0.048)
	Smoke days (overall)	-0.001 (-0.002, 0.000)	-0.001 (-0.002, 0.000)
	Smoke days (≥ 2.5 μg/m^3)	-0.001 (-0.003, 0.001)	-0.001 (-0.003, 0.001)
	Smoke days (≥5 μg/m^3)	-0.002 (-0.004, 0.001)	-0.002 (-0.004, 0.001)
	Smoke days (≥10 μg/m^3)	-0.001 (-0.007, 0.004)	-0.001 (-0.006, 0.005)
SGA	Average wildfire $PM_{2.5}$	1.051 (0.937, 1.179)	1.058 (0.942, 1.189)
	Smoke days (overall)	1.002 (0.997, 1.006)	1.002 (0.998, 1.007)
	Smoke days (≥ 2.5 μg/m^3)	1.002 (0.996, 1.008)	1.003 (0.997, 1.009)
	Smoke days (≥5 μg/m^3)	1.005 (0.996, 1.013)	1.006 (0.997, 1.015)
	Smoke days (≥10 μg/m^3)	1.007 (0.991, 1.024)	1.009 (0.992, 1.026)
LGA	Average wildfire $PM_{2.5}$	1.055 (0.949, 1.173)	1.076 (0.966, 1.199)
	Smoke days (overall)	0.999 (0.995, 1.003)	0.999 (0.995, 1.003)
	Smoke days (≥ 2.5 μg/m^3)	1.000 (0.995, 1.005)	1.000 (0.995, 1.005)
	Smoke days (≥5 μg/m^3)	1.000 (0.992, 1.008)	1.000 (0.992, 1.009)
	Smoke days (≥10 μg/m^3)	1.008 (0.992, 1.024)	1.010 (0.994, 1.027)

Note: Primary model was adjusted for maternal age, race, education, and ethnicity; child sex; neighborhood poverty rate; season of conception, year of birth splines (4 df), spatial splines (10 df), and cohort random intercept. Extended model additionally included parity, pre-pregnancy BMI, self-reported pregnancy tobacco use, and self-reported pregnancy alcohol consumption.

Table S5. Results of sensitivity analyses. Estimates represent coefficient and 95% CI for term birth weight and BWGA z-scores and OR and 95% CI for SGA and LGA.

Outcome	Adjustment	Average wildfire $PM_{2.5}$ (per ug/m^3)	Smoke days (per additional day)
Term BW	Conception month	6.7 (-9.4, 22.9)	-0.3 (-0.9, 0.3)
	Date of conception spline	14.9 (-3.6, 33.3)	-0.01 (-0.8, 0.8)
	Temperature	6.7 (-9.4, 22.8)	-0.3 (-0.9, 0.4)
	Total ambient $PM_{2.5}$	4.9 (-13.0, 22.8)	-0.2 (-0.9, 0.4)
	Site random effect	5.8 (-14.1, 25.6)	-0.2 (-0.8, 0.4)
	No site adjustment	9.3 (-6.6, 25.1)	-0.1 (-0.7, 0.5)
	Without spatial splines	8.7 (-7.2, 24.5)	-0.1 (-0.7, 0.5)
	Complete case analysis	9.2 (-7.9, 26.3)	-0.2 (-0.8, 0.4)
BWGA z-scores	Conception month	0.002 (-0.035, 0.039)	-0.001 (-0.003, 0.000)
	Date of conception spline	0.015 (-0.027, 0.057)	-0.001 (-0.003, 0.001)
	Temperature	0.005 (-0.032, 0.042)	-0.001 (-0.002, 0.000)
	Total ambient $PM_{2.5}$	0.008 (-0.033, 0.049)	-0.001 (-0.002, 0.000)
	Site random effect	0.001 (-0.045, 0.046)	-0.001 (-0.002, 0.001)
	No site adjustment	0.007 (-0.029, 0.043)	-0.001 (-0.002, 0.001)
	Without spatial splines	0.008 (-0.028, 0.044)	-0.001 (-0.002, 0.001)
	Complete case analysis	0.011 (-0.028, 0.050)	-0.001 (-0.002, 0.000)
	Exposure period: 0-32 weeks	0.004 (-0.029, 0.038)	0.000 (-0.002, 0.001)
SGA	Conception month	1.047 (0.932, 1.176)	1.001 (0.997, 1.006)
	Date of conception spline	1.049 (0.919, 1.197)	1.002 (0.997, 1.008)
	Temperature	1.050 (0.935, 1.179)	1.002 (0.997, 1.006)
	Total ambient PM2.5	1.016 (0.893, 1.155)	1.001 (0.997, 1.005)
	Site random effect	1.045 (0.903, 1.209)	1.002 (0.997, 1.008)
	No site adjustment	1.051 (0.937, 1.179)	1.002 (0.997, 1.008)
	Without spatial splines	1.047 (0.935, 1.172)	1.001 (0.997, 1.005)
	Complete case analysis	1.048 (0.933, 1.179)	1.003 (0.999, 1.007)
	Exposure period: 0-32 weeks	1.047 (0.942, 1.164)	1.001 (0.997, 1.006)
LGA	Conception month	1.044 (0.938, 1.162)	0.998 (0.994, 1.002)
	Date of conception spline	1.095 (0.969, 1.238)	0.999 (0.994, 1.004)
	Temperature	1.061 (0.954, 1.180)	1.000 (0.996, 1.004)
	Total ambient PM2.5	1.012 (0.899, 1.139)	0.998 (0.995, 1.002)
	Site random effect	1.055 (0.950, 1.173)	1.000 (0.995, 1.004)
	No site adjustment	1.055 (0.950, 1.173)	0.999 (0.995, 1.003)
	Without spatial splines	1.062 (0.956, 1.179)	1.000 (0.996, 1.003)
	Complete case analysis	1.082 (0.969, 1.208)	1.000 (0.996, 1.004)
	Exposure period: 0-32 weeks	1.041 (0.944, 1.148)	1.000 (0.996, 1.004)

Table S6. Results of trimester-specific analyses

Outcome	Trimester	Average wildfire $PM_{2.5}$ (per ug/m^3)	Smoke days (per additional day)
Term BW	First	8.3 (-2.7, 19.3)	0.4 (-0.5, 1.2)
	Second	1.6 (-7.5, 10.6)	-0.6 (-1.5, 0.3)
	Third	4.2 (-0.3, 8.8)	0.4 (-1.7, 2.5)
BWGA z-scores	First	0.020 (-0.004, 0.045)	0.001 (-0.001, 0.003)
	Second	-0.008 (-0.029, 0.012)	-0.002 (-0.004, 0.000)
	Third	0.005 (-0.006, 0.015)	0.001 (-0.004, 0.005)
SGA	First	0.971 (0.891, 1.058)	0.999 (0.992, 1.006)
	Second	1.044 (0.982, 1.110)	1.003 (0.997, 1.010)
	Third	0.988 (0.951, 1.026)	0.996 (0.980, 1.012)
LGA	First	1.066 (0.999, 1.138)	1.004 (0.998, 1.010)
	Second	0.974 (0.912, 1.039)	0.996 (0.990, 1.002)
	Third	1.018 (0.990, 1.047)	1.002 (0.988, 1.016)

Table S7. ECHO Cohort Consortium Collaborators

Name	Institution	ECHO Cohort Study Site or Core Name and Grant Number
Brian P. Smith	Duke Clinical Research Institute, Duke University School of Medicine	U2COD023375 (Coordinating Center)
Kristin L. Newby		
Linda Adair	Gillings School of Global Public Health, University of North Carolina at Chapel Hill	U2COD023375 (Coordinating Center)
Lisa P. Jacobson	Johns Hopkins University, Bloomberg School of Public Health	U24OD023382 (Data Analysis Center)
Diane Catellier	Research Triangle Institute	
Monica McGrath	Johns Hopkins University, Bloomberg School of Public Health	
Christian Douglas	Research Triangle Institute	
Priya Duggal	Johns Hopkins University, Bloomberg School of Public Health	
Emily Knapp		
Amii Kress		
Courtney K. Blackwell	Feinberg School of Medicine, Northwestern University	U24OD023319 with co-funding from the Office of Behavioral and Social Science Research (Measurement Core)
Maxwell A. Mansolf		
Jin-Shei Lai		
Emily Ho		
David Cella		
Richard Gershon		
Michelle L. Macy	Feinberg School of Medicine, Northwestern University and Ann & Robert H. Lurie Children's Hospital of Chicago	
Suman R. Das	Vanderbilt University Medical Center	U24OD035523 (Lab Core)
Jane E. Freedman		
Simon A. Mallal		
John A. McLean		
Ravi V. Shah		
Meghan H. Shilts		
Akram N. Alshawabkeh	Northeastern University	UG3/UH3OD023251 (Akram Alshawabkeh)
Jose F. Cordero	University of Georgia	
John Meeker	University of Michigan	
Leonardo Trasande	NYU Grossman School of Medicine	UG3/UH3OD023305 (Leonardo Trasande)
Carlos A. Camargo	Massachusetts General Hospital, Harvard Medical School	UG3/UH3OD023253 (Carlos Camargo)
Kohei Hasegawa		
Zhaozhong Zhu		
Ashley F. Sullivan		
Dana Dabelea	University of Colorado Anschutz Medical Campus	UG3/UH3OD023248 and UG3OD035526 (Dana Dabelea)
Wei Perng		UG3/UH3OD023248 (Dana Dabelea)
Traci A. Bekelman		
Greta Wilkening		
Sheryl Magzamen	Colorado School of Public Health, Colorado State University	UG3OD035526 (Dana Dabelea)
Brianna F. Moore	University of Colorado Anschutz Medical Campus	
Anne P. Starling	University of North Carolina at Chapel Hill	
Deborah J. Rinehart	Denver Health and Hospital Authority	
Daphne Koinis Mitchell	Rhode Island Hospital, The Alpert Medical School of Brown University	UG3/UH3OD023313 (Daphne Koinis Mitchell)
Viren D'Sa		
Sean C.L. Deoni	Bill & Melinda Gates Foundation	

Hans-Georg Mueller	University of California, Davis	
Cristiane S. Duarte	Columbia University - NYSPI	UH3OD023328 (Cristiane Duarte)
Catherine Monk		
Glorisa Canino	University of Puerto Rico, School of Medicine	
Jonathan Posner	Duke University School of Medicine, Duke Psychiatry & Behavioral Sciences	
Tenneill Murray	Columbia University - NYSPI	
Claudia Lugo-Candelas		
Anne L. Dunlop	Emory University School of Medicine	UH3OD023318 (Anne Dunlop)
Patricia A. Brennan	Emory University	
Christine Hockett	Avera Research Institute; University of South Dakota School of Medicine	UG3/UH3OD023279 (Amy Elliott)
Amy Elliott		
Assiamira Ferrara	Kaiser Permanente Northern California	UG3/UH3OD023289 (Assiamira Ferrara)
Lisa A. Croen		UG3/UH3OD023342 (Kristen Lyall), UG3/UH3OD023290 (Julie Herbstman)
Monique M. Hedderson		UG3/UH3OD023289 (Assiamira Ferrara), UG3OD035540 (Monique Marie Hedderson)
John Ainsworth	University of Manchester	UG3/UH3OD023282 (James Gern)
Leonard B. Bacharier	Vanderbilt University Medical Center	
Casper G. Bendixsen	Marshfield Clinic Research Institute	
James E. Gern	University of Wisconsin School of Medicine and Public Health	UG3/UH3OD023282 (James Gern), UG3OD035509 (Anne Marie Singh)
Diane R. Gold	Brigham and Women's Hospital; Harvard Medical School	UG3/UH3OD023282 (James Gern)
Tina V. Hartert	Vanderbilt University Medical Center	UG3/UH3OD023282 (James Gern), UG3OD035516 and UG3OD035517 (Tina Hartert)
Daniel J. Jackson	University of Wisconsin School of Medicine and Public Health	UG3/UH3OD023282 (James Gern)
Christine C. Johnson	Henry Ford Health	UG3/UH3OD023282 (James Gern), UG3OD035518 (Jennifer Straughen)
Christine L.M. Joseph		UG3/UH3OD023282 (James Gern)
Meyer Kattan	Columbia University Medical Center	
Gurjit K. Khurana Hershey	Cincinnati Children's Hospital Medical Center	
Robert F. Lemanske, Jr.	University of Wisconsin School of Medicine and Public Health	
Susan V. Lynch	University of California	
Rachel L. Miller	Icahn School of Medicine at Mount Sinai	UG3/UH3OD023282 (James Gern), UG3/UH3OD023290 (Julie Herbstman)
George T. O'Connor	Boston University School of Medicine	UG3/UH3OD023282 (James Gern)
Carole Ober	University of Chicago	UG3/UH3OD023282 (James Gern), UG3OD035509 (Anne Marie Singh)
Dennis Ownby	Henry Ford Health	UG3/UH3OD023282 (James Gern)
Katherine Rivera-Spoljaric	Washington University School of Medicine	UG3/UH3OD023282 (James Gern), UG3OD035521 (Katherine Rivera-Spoljaric)
Patrick H. Ryan	University of Cincinnati	UG3/UH3OD023282 (James Gern), UG3OD035509 (Anne Marie Singh)
Christine M. Seroogy	University of Wisconsin School of Medicine and Public Health	UG3/UH3OD023282 (James Gern)
Anne Marie Singh	University of Wisconsin School of Medicine and Public Health	UG3/UH3OD023282 (James Gern), UG3OD035509 (Anne Marie Singh)
Robert A. Wood	Johns Hopkins University School of Medicine	UG3/UH3OD023282 (James Gern)
Edward M. Zoratti	Henry Ford Health	UG3/UH3OD023282 (James Gern), UG3OD035518 (Jennifer Straughen)
Rima Habre	University of Southern California	UH3OD023287 (Carrie Breton)
Shohreh Farzan		
Frank D. Gilliland		
Irva Hertz-Picciotto	University of California, Davis	UG3/UH3OD023365 (Irva Hertz-Picciotto), UG3OD035550 (Rebecca Schmidt)
Deborah H. Bennett		

Julie B. Schweitzer		UG3/UH3OD023365 (Irva Hertz-Picciotto)
Rebecca J. Schmidt		UG3/UH3OD023365 (Irva Hertz-Picciotto), UG3/UH3OD023342 (Kristen Lyall), UG3OD035550 (Rebecca Schmidt)
Janine M. LaSalle		UG3/UH3OD023365 (Irva Hertz-Picciotto), UG3OD035550 (Rebecca Schmidt)
Alison E. Hipwell	University of Pittsburgh	UG3/UH3OD023244 (Alison Hipwell)
Kate E. Keenan	University of Chicago	
Catherine J. Karr	University of Washington	UH3OD023271 and UG3OD035528 (Catherine Karr)
Nicole R. Bush	University of California, San Francisco	UH3OD023271 (Catherine Karr), UG3OD035519 (Qi Zhao)
Kaja Z. LeWinn		
Sheela Sathyanarayana	University of Washington and Seattle Children's Research Institute	UH3OD023271 (Catherine Karr), UG3OD035508 (Sheela Sathyanarayana)
Qi Zhao	University of Tennessee Health Science Center	UH3OD023271 (Catherine Karr), UG3OD035519 (Qi Zhao)
Frances Tylavsky	University of Tennessee Health Science Center	UH3OD023271 (Catherine Karr)
Kecia N. Carroll	Icahn School of Medicine at Mount Sinai	UH3OD023271 (Catherine Karr), UG3/UH3OD023337 (Rosalind Wright)
Christine T. Loftus	University of Washington	UH3OD023271 (Catherine Karr)
Leslie D. Leve	University of Oregon	UG3/UH3OD023389 (Leslie Leve)
Jody M. Ganiban	George Washington University	
Jenae M. Neiderhiser	Penn State University	
Scott T. Weiss	Brigham and Women's Hospital and Harvard Medical School	UH3OD023268 (Scott Weiss)
Augusto A. Litonjua	Golisano Children's Hospital, University of Rochester	
Cindy T. McEvoy	Oregon Health & Science University	UG3/UH3OD023288 (Cynthia McEvoy)
Eliot R. Spindel	Oregon National Primate Research Center	
Robert S. Tepper	Indiana School of Medicine	
Craig J. Newschaffer	Penn State	UG3/UH3OD023342 (Kristen Lyall)
Kristen Lyall	Drexel University	
Heather E. Volk	Johns Hopkins University	
Rebecca Landa	Center for Autism and Related Disorders, Kennedy Krieger Institute, Johns Hopkins University	
Sally Ozonoff	University of California Davis	
Joseph Piven	University of North Carolina	
Heather Hazlett	University of North Carolina	
Juhi Pandey	Children's Hospital of Philadelphia	
Robert Schultz	Children's Hospital of Philadelphia	
Steven Dager	University of Washington	
Kelly Botteron	Washington University	
Daniel Messinger	University of Miami	
Wendy Stone	University of Washington	
Jennifer Ames	Kaiser Permanente	
Thomas G. O'Connor	University of Rochester	UG3/UH3OD023349 (Thomas O'Connor)
Richard K. Miller		
Emily Oken	Harvard Pilgrim Health Care Institute and Harvard Medical School	UH3OD023286 and UG3OD035533 (Emily Oken)
Michele R. Hacker	Beth Israel Deaconess Medical Center	UG3OD035533 (Emily Oken)
Tamarra James-Todd	Harvard Chan School of Public Health	UG3OD035533 (Emily Oken)
Michael O'Shea	University of North Carolina School of Medicine	UG3/UH3OD023348 (Mike O'Shea), UH3OD023347 (Barry Lester)
Rebecca C. Fry	University of North Carolina Gillings School of Global Public Health	UG3/UH3OD023348 (Mike O'Shea)
Jean A. Frazier	UMASS Chan Medical School	

Rachana Singh	Tufts University School of Medicine	
Caitlin Rollins	Harvard Medical School	
Angela Montgomery	Yale School of Medicine	
Ruben Vaidya	University of Massachusetts Chan Medical School-Baystate	
Robert M. Joseph	Boston University Chobanian & Avedisian School of Medicine	
Lisa K. Washburn	Wake Forest School of Medicine	
Semsa Gogcu	Wake Forest School of Medicine; Wake Forest University School of Medicine/Atrium Health Wake Forest	UG3/UH3OD023348 (Mike O'Shea), UG3OD035513 (Annemarie Stroustrup), UH3OD023320 (Judy Aschner)
Kelly Bear	ECU Health	UG3/UH3OD023348 (Mike O'Shea)
Julie V. Rollins	University of North Carolina School of Medicine	
Stephen R. Hooper		
Genevieve Taylor		
Wesley Jackson		
Amanda Thompson	University of North Carolina at Chapel Hill; Gillings School of Global Public Health, University of North Carolina at Chapel Hill	
Julie Daniels		
Michelle Hernandez	School of Medicine, University of North Carolina at Chapel Hill	
Kun Lu	Gillings School of Global Public Health, University of North Carolina at Chapel Hill	
Michael Msall	University of Chicago Medicine: Comer Children's Hospital	
Madeleine Lenski	Michigan State University	
Rawad Obeid	Beaumont Hospital	
Steven L. Pastyrnak	Corewell Health, Helen DeVos Children's Hospital	UG3/UH3OD023348 (Mike O'Shea), UH3OD023347 (Barry Lester)
Elizabeth Jensen	Wake Forest University School of Medicine	UG3/UH3OD023348 (Mike O'Shea)
Christina Sakai	Mass General Hospital for Children	UG3/UH3OD023348 (Mike O'Shea)
Hudson Santos	University of Miami	UG3/UH3OD023348 (Mike O'Shea), UG3OD035542 (Hudson Santos)
Jean M. Kerver	Michigan State University, College of Human Medicine	UG3/UH3OD023285 (Jean Kerver)
Nigel Paneth		UG3/UH3OD023285 (Jean Kerver)
Charles J. Barone	Henry Ford Health	UG3/UH3OD023285 (Jean Kerver), UG3/UH3OD023282 (James Gern)
Michael R. Elliott	University of Michigan	UG3/UH3OD023285 (Jean Kerver)
Douglas M. Ruden	Wayne State University	UG3/UH3OD023285 (Jean Kerver)
Chris Fussman	Michigan Department of Health and Human Services (MDHHS)	UG3/UH3OD023285 (Jean Kerver)
Julie B. Herbstman	Columbia University Mailman School of Public Health	UG3/UH3OD023290 (Julie Herbstman)
Amy Margolis	Columbia University Irving Medical Center	UG3/UH3OD023290 (Julie Herbstman)
Susan L. Schantz	University of Illinois Urbana-Champaign	UG3/UH3OD023272 (Susan Schantz)
Sarah Dee Geiger		
Andrea Aguiar		
Karen Tabb		
Rita Strakovsky	Michigan State University	
Tracey Woodruff	University of California, San Francisco	
Rachel Morello-Frosch	University of California, Berkeley	
Amy Padula	University of California, San Francisco	
Joseph B. Stanford	Spencer Fox Eccles School of Medicine, University of Utah	UG3/UH3OD023249 (Joseph Stanford)
Christina A. Porucznik		
Angelo P. Giardino		
Rosalind J. Wright	Icahn School of Medicine at Mount Sinai	UG3/UH3OD023337 (Rosalind Wright)
Robert O. Wright		

Brent Collett	University of Washington, Seattle Children's Research Institute	UG3OD035508 (Sheela Sathyanarayana)
Nicole Baumann-Blackmore	University of Wisconsin School of Medicine and Public Health	UG3OD035509 (Anne Marie Singh)
Ronald Gangnon	University of Wisconsin	
Daniel J. Jackson	University of Wisconsin School of Medicine and Public Health	
Chris G. McKennan	University of Pittsburgh	
Jo Wilson	University of Wisconsin School of Medicine and Public Health	
Matt Altman	University of Washington	
Judy L. Aschner	Albert Einstein College of Medicine; Hackensack Meridian School of Medicine; Center for Discovery and Innovation	UH3OD023320 and UG3OD035546 (Judy Aschner), UG3OD035513 (Annemarie Stroustrup)
Annemarie Stroustrup	Northwell Health, Cohen Children's Medical Center, and the Zucker School of Medicine at Hofstra / Northwell	UH3OD023320 (Judy Aschner), UG3OD035513 (Annemarie Stroustrup)
Stephanie L. Merhar	Cincinnati Children's	
Paul E. Moore	Vanderbilt University Medical Center	
Gloria S. Pryhuber	University of Rochester Medical Center	UH3OD023320 (Judy Aschner)
Mark Hudak	University of Florida College of Medicine	
Ann Marie Reynolds Lyndaker	University of Buffalo Jacobs School of Medicine and Biomedical Sciences	
Andrea L. Lampland	Children's Minnesota	
Burton Rochelson	Northwell Health and the Zucker School of Medicine at Hofstra / Northwell	UG3OD035532 (Annemarie Stroustrup)
Sophia Jan	Northwell Health, Cohen Children's Medical Center, and the Zucker School of Medicine at Hofstra / Northwell	
Matthew J. Blitz	Northwell Health and the Zucker School of Medicine at Hofstra / Northwell	
Michelle W. Katzow		
Zenobia Brown	Northwell Health and the Zucker School of Medicine at Hofstra / Northwell	
Codruta Chiuzan	Northwell Health, Feinstein Institutes for Medical Research	
Timothy Rafael	Northwell Health and the Zucker School of Medicine at Hofstra / Northwell	
Dawnette Lewis		
Natalie Meirowitz		
Brenda Poindexter	Children's Healthcare of Atlanta Emory University	UH3OD023320 (Judy Aschner)
Tebeb Gebretsadik	Vanderbilt University Medical Center	UG3OD035516 and UG3OD035517 (Tina Hartert)
Sarah Osmundson	Vanderbilt University Medical Center	UG3OD035517 (Tina Hartert)
Jennifer K. Straughen	Henry Ford Health	UG3OD035518 (Jennifer Straughen)
Amy Eapen		
Andrea Cassidy-Bushrow		UG3/UH3OD023282 (James Gern)
Ganesa Wegienka		
Alex Sitarik		
Kim Woodcroft		UG3OD035518 (Jennifer Straughen), UG3/UH3OD023282 (James Gern)
Audrey Urquhart		
Albert Levin		UG3OD035518 (Jennifer Straughen)
Tisa Johnson-Hooper		
Brent Davidson		UG3/UH3OD023282 (James Gern)
Tengfei Ma		UG3OD035518 (Jennifer Straughen)
Emily S. Barrett	Environmental and Occupational Health Sciences Institute, Rutgers University	UG3OD035527 (Emily S Barrett)
Martin J. Blaser	Rutgers University	
Maria Gloria Dominguez-Bello		
Daniel B. Horton	Robert Wood Johnson Medical School, Rutgers University	
Manuel Jimenez		

Todd Rosen		
Kristy Palomares	Saint Peter's University Hospital	
Lyndsay A. Avalos	Kaiser Permanente Northern California	UG3OD035540 (Monique Marie Hedderson)
Yeyi Zhu		
Kelly J . Hunt	Medical University of South Carolina	UG3OD035543 (Kelly J Hunt)
Roger B. Newman	Medical University of South Carolina	
Michael S. Bloom	George Mason University	
Mallory H. Alkis	Medical University of South Carolina	
James R. Roberts	Medical University of South Carolina	
Sunni L. Mumford	University of Pennsylvania Perelman School of Medicine	UG3OD035537 (Sunni L Mumford)
Heather H. Burris	Children's Hospital of Philadelphia; University of Pennsylvania Perelman School of Medicine	
Sara B. DeMauro		
Lynn M. Yee	Feinberg School of Medicine, Northwestern University	UG3OD035546 (Judy Aschner)
Aaron Hamvas	Ann & Robert H. Lurie Children's Hospital, Feinberg School of Medicine, Northwestern University	
Antonia F. Olidipo	Hackensack University Medical Center, Hackensack Meridian School of Medicine	
Andrew S. Haddad		
Lisa R. Eiland		
Nicole T. Spillane		
Kirin N. Suri		
Stephanie A. Fisher	Feinberg School of Medicine, Northwestern University	
Jeffrey A. Goldstein	Feinberg School of Medicine, Northwestern University	
Leena B. Mithal	Ann & Robert H. Lurie Children's Hospital, Feinberg School of Medicine, Northwestern University	
Raye-Ann O. DeRegnier	Ann & Robert H. Lurie Children's Hospital, Feinberg School of Medicine, Northwestern University	
Nathalie L. Maitre	Emory University School of Medicine and Cerebral Palsy Foundation	
Ruby H.N. Nguyen	School of Public Health, University of Minnesota	UG3OD035529 (Hong-Ngoc Nguyen)
Meghan M. JaKa	HealthPartners Institute	
Abbey C. Sidebottom	Allina Health	
Michael J. Paidas	University of Miami Miller School of Medicine	UG3OD035542 (Hudson Santos)
JoNell E. Potter		
Natale Ruby		
Lunthita Duthely		
Arumugam Jayakumar		
Karen Young		
Isabel Maldonado	University of Miami	
Meghan Miller	University of California Davis	UG3OD035550 (Rebecca Schmidt)
Jonathan L. Slaughter	Nationwide Children's Hospital and The Ohio State University	UG3OD035536 (Jonathan Slaughter)
Sarah A. Keim		
Courtney D. Lynch	The Ohio State University	
Kartik K. Venkatesh		
Kristina W. Whitworth	Baylor College of Medicine	UG3OD035544 (Kristina Whitworth)
Elaine Symanski		
Thomas F. Northrup	University of Texas Health Science Center at Houston (UTHealth Houston) McGovern Medical School	
Hector Mendez-Figueroa		
Ricardo A. Mosquera		
Margaret R. Karagas	Geisel School of Medicine at Dartmouth	UG3/UH3OD023275 (Margaret Karagas)

Juliette C. Madan	Geisel School of Medicine at Dartmouth, Dartmouth Hitchcock Medical Center	
Debra M. MacKenzie	College of Pharmacy, University of New Mexico Health Sciences Center	UG3/UH3OD023344 (Debra MacKenzie)
Johnnye L. Lewis		
Brandon J. Rennie	University of New Mexico	
Bennett L. Leventhal	College of Pharmacy, University of New Mexico Health Sciences Center; University of Chicago	
Young Shin Kim	University of California, San Francisco	
Somer Bishop	University of California, San Francisco	
Sara S. Nozadi	College of Pharmacy, University of New Mexico Health Sciences Center	
Li Luo	Comprehensive Cancer Center, University of New Mexico Health Sciences Center	
Barry M. Lester	Warren Alpert Medical School of Brown University	UH3OD023347 (Barry Lester)
Carmen J. Marsit	Rollins School of Public Health, Emory University	
Todd Everson		
Cynthia M. Loncar	Warren Alpert Medical School of Brown University	
Elisabeth C. McGowan		
Stephen J. Sheinkopf	Thompson Center for Autism & Neurodevelopment, University of Missouri	
Brian S. Carter	Children's Mercy-Kansas City	
Jennifer Check	Wake Forest School of Medicine	
Jennifer B. Helderman		
Charles R. Neal	University of Hawaii John A Burns School of Medicine	
Lynne M. Smith	UCLA Clinical and Translational Science Institute at The Lundquist Institute, Harbor-UCLA Medical Center	

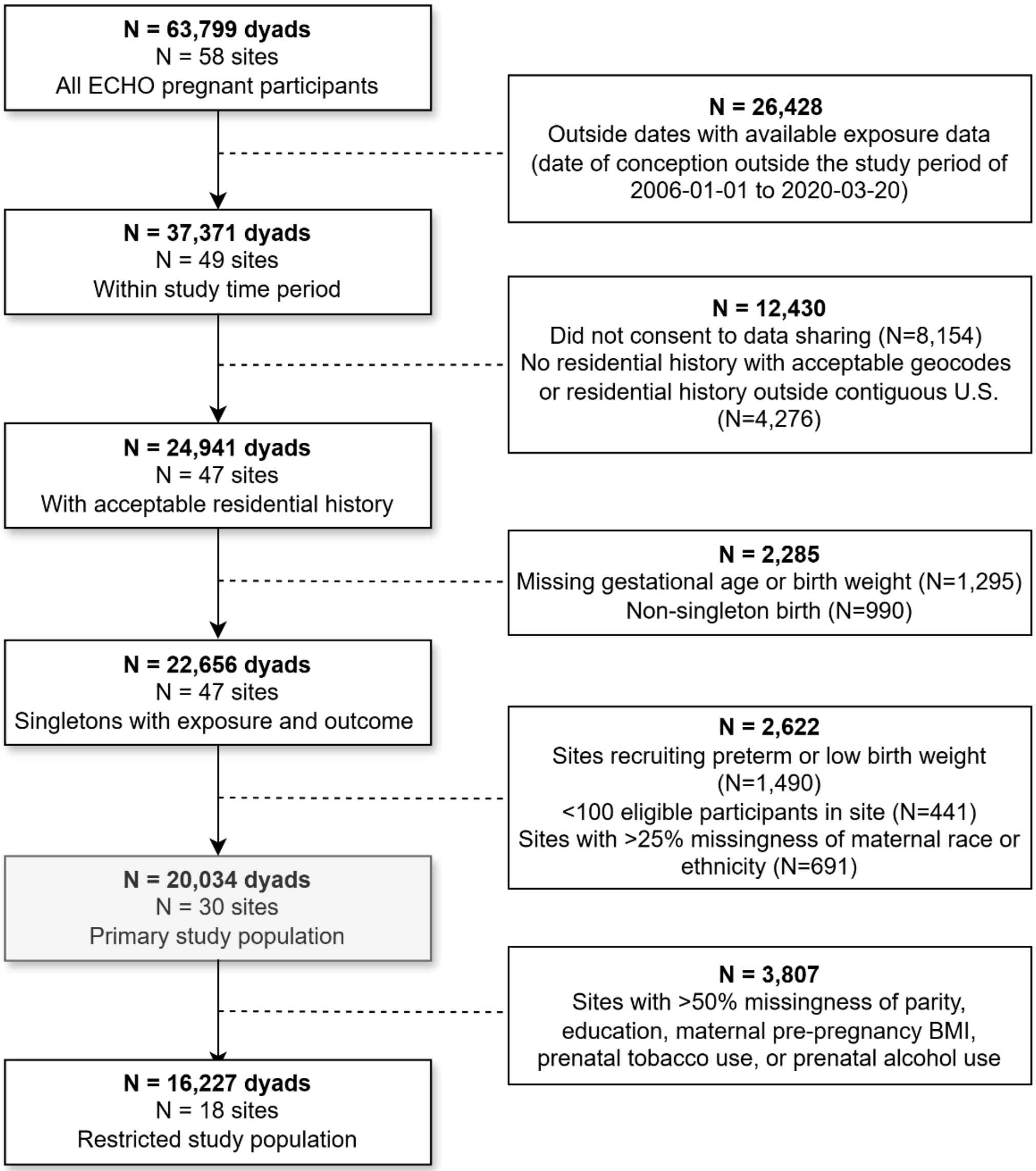

Figure S1. Inclusion flowchart. Reproduced with permission from Sherris et al. (2025)

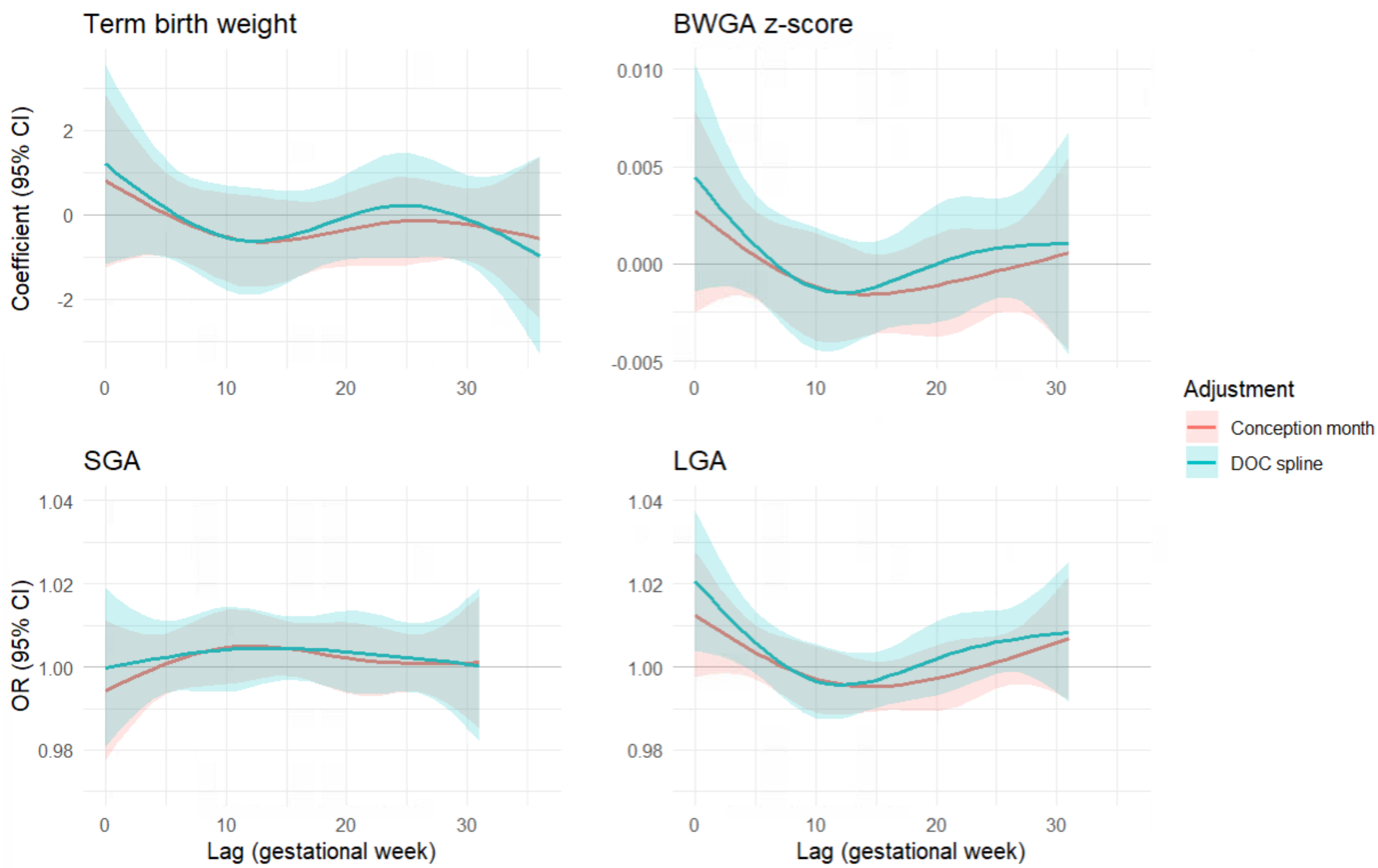

Figure S2. Results of distributed lag models with more flexible seasonal adjustment

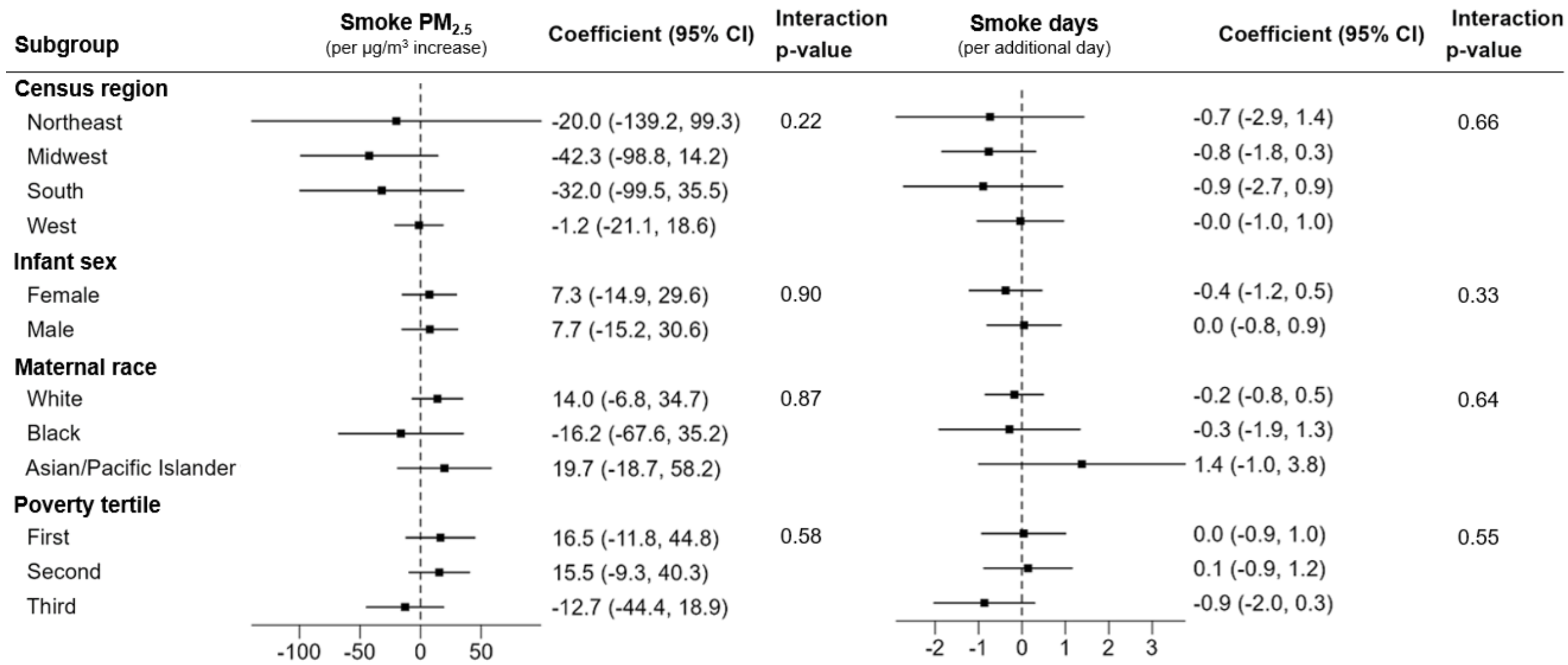

Figure S3. Effect modification in the relationship between wildfire $PM_{2.5}$ exposure and birth weight at term (g).
